# Transcription factor SOX4 promotes proliferation, invasion and lymphatic metastasis of laryngeal squamous cell carcinoma via PTBP2 activation

**DOI:** 10.3389/fonc.2026.1829851

**Published:** 2026-05-21

**Authors:** Deli Kong, Jun Hao, Jianwang Yang, Yunchao Xin, Huan Cao, Qiuli Li, Binglu He, Baoshan Wang

**Affiliations:** 1 Department of Otolaryngology-Head and Neck Surgery, The Second Hospital of Hebei Medical University, Shijiazhuang, China; 2 Department of Pathology, Hebei Medical University, Shijiazhuang, China; 3 Center of Metabolic Diseases and Cancer Research, Institute of Medical and Health Science of Hebei Medical University, Shijiazhuang, China; 4 Hebei Key Laboratory of Forensic Medicine, Shijiazhuang, China; 5 The First Affiliated Hospital of Hebei North University, Zhangjiakou, China; 6 The Fourth Hospital of Hebei Medical University, Shijiazhuang, China

**Keywords:** laryngeal squamous cell carcinoma, lymph node metastasis, PTBP2, SOX4, tumor progression

## Abstract

**Background:**

Laryngeal squamous cell carcinoma (LSCC) is a common malignant tumor in otorhinolaryngology, with a high incidence rate and a tendency of lymph node metastasis. This study aims to systematically evaluate the expression of the transcription factor SOX4 in LSCC and its clinical relevance, clarify its impact on the biological behavior of tumors and potential molecular mechanisms, and provide a theoretical basis for diagnosis and treatment.

**Method:**

Bioinformatics analysis was conducted based on the GEO dataset (GSE201777) to evaluate the expression of SOX4 and its clinical relevance; The expression of SOX4 in LSCC clinical tissues and cell lines was verified by qRT-PCR. SOX4 knockdown and overexpression cell models were constructed. Proliferation was evaluated by MTS and colony formation assays, migration and invasion were evaluated by scratch and Transwell assays, and apoptosis was detected by flow cytometry and TUNEL. The downstream effector molecules of SOX4 were screened by transcriptome sequencing and verified by functional recovery experiments. The association between SOX4 and tumor immune infiltration as well as drug sensitivity was explored by combining bioinformatics analysis. Ultimately, the effects of SOX4 on tumor growth and lymph node metastasis *in vivo* were evaluated in a xenograft mouse model.

**Result:**

Bioinformatics and experimental data consistently showed that SOX4 was upregulated in LSCC and positively correlated with disease progression and lymph node metastasis. *In vitro* experiments have shown that knockdown of SOX4 significantly inhibits the proliferation, migration and invasion of LSCC cells, while overexpression of SOX4 promotes a malignant phenotype. Transcriptome and functional verification determined that PTBP2 is a downstream effector molecule of SOX4. The restored expression of PTBP2 can partially alleviate the inhibitory effect caused by SOX4 knockdown. Bioinformatics analysis further suggests that the SOX4/PTBP2 axis is associated with the tumor immune microenvironment and the sensitivity to several anti-cancer drugs. *In vivo* studies have shown that knockdown of SOX4 or administration of erlotinib significantly inhibited tumor growth and reduced the rate of lymph node metastasis.

**Conclusion:**

SOX4 promotes the growth and lymph node metastasis of LSCC by regulating PTBP2. The SOX4-PTBP2 axis may become a potential diagnostic and therapeutic target for LSCC.

## 1 Introduction

Laryngeal squamous cell carcinoma (LSCC) is one of the most common subtypes of head and neck squamous cell carcinoma and represents a major category of malignancies in otorhinolaryngology. According to age-standardized data released in 2026 by the World Health Organization (WHO) Global Cancer Observatory (GCO), the incidence of LSCC accounts for approximately 1%–5% of all malignancies, and its mortality ranks 18th worldwide. Within the spectrum of otorhinolaryngological tumors, the incidence of LSCC is second only to nasopharyngeal carcinoma and has shown a continuous upward trend globally (Tang et al., 2019) (1) Although comprehensive treatment strategies—including surgery, radiotherapy, chemotherapy, and targeted therapy—have been progressively optimized in recent years, the overall prognosis of patients with LSCC remains unsatisfactory due to its highly invasive nature and strong propensity for regional lymph node metastasis (Gimm et al., 2025) (2). Therefore, systematically elucidating the mechanisms underlying LSCC initiation and progression, particularly the molecular basis of lymph node metastasis, and identifying reliable diagnostic biomarkers and potential therapeutic targets are of great theoretical and clinical significance.

During tumor initiation and progression, transcription factors play central regulatory roles by controlling key gene expression networks. Among them, the SRY-related high-mobility group box (SOX) family of transcription factors has attracted considerable attention because of its crucial involvement in embryonic development, cell fate determination, and maintenance of tissue homeostasis (Liu et al., 2021) (3). SOX4, an important member of the SOX family, encodes a transcription factor containing an HMG-box domain that can specifically bind to promoter or enhancer regions of target genes, thereby precisely regulating downstream gene transcription (Wang et al., 2025) (4). Previous studies have demonstrated that SOX4 is aberrantly overexpressed in multiple malignancies, including hepatocellular carcinoma, colorectal cancer, and lung cancer, and is closely associated with enhanced tumor cell proliferation, activation of epithelial–mesenchymal transition (EMT), and increased migratory and invasive capacities (Li et al., 2024; Tan et al., 2021; Luo et al., 2021) (5–7).

In LSCC, existing evidence suggests that SOX4 may be involved in tumor initiation and progression; however, its specific functional role and molecular regulatory mechanisms in lymph node metastasis have not yet been systematically elucidated (Zhang et al., 2025) (8). In addition to tumor cell–intrinsic regulatory networks, the tumor immune microenvironment has been recognized as a critical determinant of tumor biological behavior and therapeutic response (Fernandes et al., 2023) (9). Recent studies have further revealed that SOX4 not only directly regulates tumor cell phenotypes but also indirectly participates in tumor progression and modulation of treatment sensitivity by influencing immune cell infiltration, expression of immune-related genes, and drug-response pathways (Deng et al., 2023; Wang et al., 2023) (10, 11). Nevertheless, whether SOX4 cooperatively regulates the tumor immune microenvironment and drug responsiveness through downstream effector molecules in LSCC remains largely unexplored.

Based on this background, the present study first screens and validates the expression profile of SOX4 in LSCC and its association with lymph node metastasis. Subsequently, the effects of SOX4 on LSCC cell proliferation, migration, invasion, and apoptosis are systematically evaluated. Furthermore, potential downstream effector molecules of SOX4 are identified through transcriptome sequencing, and their key roles in regulating malignant phenotypes of LSCC are verified. Finally, bioinformatics approaches are integrated to predict and analyze the relationships between SOX4 and its downstream genes with tumor immune infiltration characteristics and drug sensitivity. This study aims to elucidate the potential molecular mechanisms by which SOX4 contributes to LSCC progression and lymph node metastasis, and to provide experimental evidence and theoretical support for SOX4 and its downstream molecules as potential diagnostic biomarkers and therapeutic targets for LSCC.

## 2 Materials and methods

### 2.1 Clinical samples

This study collected tissue specimens from patients with laryngeal squamous cell carcinoma (LSCC) who underwent surgical treatment in the Department of Otorhinolaryngology of the Second Hospital of Hebei Medical University from December 2015 to December 2019. A total of 58 pairs of LSCC tumor tissues and adjacent non-tumor tissues were collected. None of the patients received radiotherapy or chemotherapy before the operation. All tissue specimens were independently diagnosed and confirmed by two experienced clinical pathologists. Fresh tissue specimens should be immediately quick-frozen in liquid nitrogen and stored at -80 °C for future use. All procedures for the collection of human specimens were approved by the Ethics Committee of the Second Hospital of Hebei Medical University, and all subjects signed written informed consent forms.

### 2.2 Cell culture

The human laryngeal squamous cell carcinoma cell lines TU177 and AMC-HN-8, as well as the human nasopharyngeal epithelial cell lines NP69 and HEK293T cell lines, were all preserved in the Biobank of the Department of Otorhinolaryngology Head and Neck Surgery of Hebei Medical University. TU177 cells were cultured in RPMI-1640 medium (Gibco, Shanghai, China) containing 10% fetal bovine serum (FBS, Lonsera, Uruguay); AMC-HN-8 and HEK293T cells were cultured in DMEM medium containing 10% FBS (Gibco, Shanghai, China); NP69 cells were cultured in DMEM medium. NP69, an immortalized non-malignant human nasopharyngeal epithelial cell line, was used as the normal epithelial control because matched normal laryngeal epithelial cell lines were not readily available in our biobank. All cells were cultured in a constant temperature and humidity incubator at 37 °C and 5% CO_2_.

### 2.3 RNA extraction and quantitative real-time PCR (qRT-PCR)

According to the instructions, total RNA was extracted from tissues or cells using RNAeasy™ Isolation Reagent (Vazyme, Nanjing, China). The reverse transcription reaction was performed using the first-stranded cDNA synthesis kit (Roche Diagnostics, Mannheim, Germany): 65 °C for 10 min, 85 °C for 5 min, and the total reaction volume was 13 μL. The resulting cDNA was stored for a short period at 4 °C or for a long period at -20 °C. qRT-PCR was performed using GoTaq^®^ qPCR Master Mix (Promega, Wisconsin, USA) on the ABI QuantStudio™ 5 real-time fluorescence quantitative PCR system (Thermo Fisher Scientific, USA). The total reaction system is 15 μL. As an internal reference gene, the relative expression level of GAPDH mRNA was calculated by the 2^-^ΔΔCt method. All primer sequences are shown in **Table 1**.

**Table 1:** Sequences of all primers used for qRT-PCR.

Primer name	Primer sequences (F: forward primer, R: reverse primer)
GAPDH	F: AGGTGAAGGTCGGAGTCAACG
	R: AGGGGTCATTGATGGCAACA
SOX4	F: AGCGACAAGATCCCTTTCATTC
	R: CGTTGCCGGACTTCACCTT
CAMKK2	F: CATGAACGGACGCTGCATCT
	R: ACAGTCCTGCATACCCGTGAT
DND1	F: AGTGTGAGCTGAGCGTTGAC
	R: CCGGTGCGAGCTGAATTTG
RPL21	F: TAAGCACTCTAAGAGCCGAGAT
	R: GCGCTTTAGTTGAACCCAGGTA
PTBP2	F: GCAACCGAGGAAGCAGCTATT
	R: GCCTGAGCACGTTGGTTTAATG
IFI6	F: GGTCTGCGATCCTGAATGGG
	R: TCACTATCGAGATACTTGTGGGT
IFI27	F: TGCTCTCACCTCATCAGCAGT
	R: CACAACTCCTCCAATCACAACT
SOD1	F: GGTGGGCCAAAGGATGAAGAG
	R: CCACAAGCCAAACGACTTCC

### 2.4 Cell transfection

The SOX4 overexpression plasmid (oeSOX4) and the siRNA targeting SOX4 and PTBP2 were purchased from Hanbio (Shanghai, China), and the PTBP2 overexpression plasmid (oePTBP2) was purchased from Youbio (Changsha, China). Wild-type PTBP2 and its siRNA binding site mutant (PTBP2-MUT) were cloned into the pLVX-GFP-PURO vector (Hanbio). Cell transfection was performed using Lipofectamine™ 2000 (Invitrogen, California, USA). siSOX4 was transfected into TU177, AMC-HN-8, HEK293T and NP69 cells; oeSOX4 was transfected into TU177 and AMC-HN-8 cells; siPTBP2 and oePTBP2 were transfected into TU177 and AMC-HN-8 cells; PTBP2-Mut was transfected into TU177 and AMC-HN-8 cells. The corresponding negative controls were siNC, oeNC or empty vectors respectively. The transfected cells were used for subsequent functional experiments.

### 2.5 MTS cell proliferation assay

After transfection for 24 h, TU177 and AMC-HN-8 cells were seeded in 96-well plates at a density of 2 × 10³ cells per well, and 100 μL of complete medium containing 10% FBS was added to each well. Then add 20 μL of MTS reagent (CellTiter 96^®^ AQueous One Solution Cell Proliferation Assay, Promega) to each well and incubate at 37 °C for 2 hours. Absorbance was measured at a wavelength of 490 nm using a Spark^®^ multifunctional microplate reader (SPARK 10 M, TECAN, Switzerland).

### 2.6 Clone formation experiment

The transfected TU177 and AMC-HN-8 cells were seeded in 6-well plates at a rate of 2000 cells per well and cultured at 37 °C for approximately 10 days. When the number of cells in a single clone is greater than 50, it is determined to be a valid clone. Cells were fixed with 4% paraformaldehyde for 20 min, stained with 0.1% crystal violet for 20 min, photographed, recorded and counted.

### 2.7 Transwell migration and invasion assay

After 24 h transfection, TU177 and AMC-HN-8 cells were collected and resuspended in serum-free medium. Then, they were inoculated in the upper chamber of Transwell (Corning, New York, USA) at a rate of 1 × 10^5^ cells/well, and 650 μL of medium containing 10% FBS was added to the lower chamber. In the invasion test, the upper chamber was pre-coated with Matrigel (Corning). After incubation for 24 h, the non-migrating cells in the upper chamber were gently wiped off with cotton swabs, and the cells in the lower chamber were fixed with 4% paraformaldehyde for 20 min and stained with 0.1% crystal violet for 20 min, and photographed under an optical microscope. The experiment was repeated twice. These migration and invasion assays were completed within a short experimental window after transfection and were interpreted together with the proliferation and apoptosis data to minimize potential confounding from altered cell growth on motility readouts.

### 2.8 Scratch healing test

The transfected TU177 and AMC-HN-8 cells were seeded in 6-well plates. When the cell fusion was close to 100%, a 200 μL pipette tip was used to make vertical scratches on the monolayer cell surface. Photos were taken at 0, 24, 48 and 72 h respectively. The scratch area was measured using ImageJ software, and the relative healing rate was calculated: The relative scratch healing rate = (0 h scratch area - scratch area at the corresponding time point)/0 h scratch area. To further reduce proliferation-related confounding, the scratch assay was analyzed in parallel with the MTS and apoptosis results, and the observation window was kept short.

### 2.9 Apoptosis was detected by flow cytometry

Apoptosis was detected using the Annexin V-FITC/PI apoptosis detection kit (Servicebio, Wuhan, China). AMC-HN-8 cells were collected at 24 h after transfection, and TU177 cells were collected at 24 h and 48 h after transfection. Double staining of Annexin V-FITC and PI was performed as per the instructions, and the proportion of apoptosis was detected and analyzed using CytoFLEX LX flow cytometry (Beckman Coulter, China).

### 2.10 TUNEL apoptosis detection

The transfected TU177 and AMC-HN-8 cells were seeded into 6-well plates containing 24 mm cell slides. When the cell fusion was approximately 70%, they were fixed with 4% paraformaldehyde for 20 min and permeated with 0.2% Triton X-100 for 5 min. Subsequently, staining was performed using TUNEL BrightRed Apoptosis Detection Kit (Vazyme). The plates were encapsulated with an anti-fluorescence quenching mounting agent containing DAPI and observed and photographed under a laser confocal microscope (Leica, Germany).

### 2.11 Bioinformatics analysis and clinical correlation analysis

The analysis was conducted using the GSE201777 dataset (GPL15027 platform) in the GEO database. After excluding non-tumor samples, LSCC tissues were divided into the metastasis-positive group and the metastasis-negative group according to the presence or absence of regional lymph node metastasis. Differential expression analysis was performed using R Studio 4.3.1, and functional enrichment analysis was conducted using GO, KEGG and GSEA. The protein-protein interaction network was constructed through the STRING database, and the key genes were screened by using the maximum group centrality (MCC) algorithm in Cytoscape 3.10.2. Further, the TCGA, TIMER2.0 and DepMap databases were combined to analyze the gene correlation, survival prognosis and immune infiltration characteristics.

### 2.12 Cell transcriptome sequencing

The expression of SOX4 in TU177 cells was knocked down by siRNA, and the cells were collected after transfection for 24 h. RNA was extracted by TRIzol lysis, and the cells were rapidly frozen in liquid nitrogen and stored at -80 °C. The samples were sent to Shanghai Meiji Biomedical Technology Co., Ltd. for RNA sequencing and subsequent bioinformatics analysis.

### 2.13 Xenograft tumor formation and lymph node metastasis model in nude mice

Construct a TU177 cell line with stable knockdown of SOX4. 4-5-week-old SPF-grade male BALB/c nude mice (Beijing Weitonglihua Laboratory Animal Technology Co., LTD.) were selected and raised at the Laboratory Animal Public Service Platform of Hebei Medical University. Subcutaneous tumor formation model: Subcutaneously inject 5 × 10^6^ cells/100 μL PBS behind the right scapula; Lymph node metastasis model: Inject 2 × 10^6^ cells/50 μL PBS into the left foot pad. When the tumor volume was ≥100 mm³, some mice were intraperitoneally injected with erlotinib (20 mg/kg, q3d). The formula for calculating tumor volume: V = (length × width²)/2. After experiment 28 days, the mice were sacrificed and samples were collected. At the experimental endpoint (day 28), mice were anesthetised prior to euthanasia. Specifically, animals were anaesthetised with intraperitoneal pentobarbital sodium (120 mg/kg) until deep anaesthesia was achieved (loss of pedal withdrawal and palpebral reflexes). Euthanasia was performed by cervical dislocation while animals were deeply anaesthetised, and death was confirmed by cessation of respiration and heartbeat. All procedures were performed to minimise pain and distress and were carried out in accordance with the approved institutional protocol (Animal Care and Ethics Committee of Hebei Medical University; IACUC No. IACUC-Hebmu-P2022101). All animal experiments were approved by the Ethics Committee of the Second Hospital of Hebei Medical University.

### 2.14 Statistical analysis

Measurement data were expressed as mean ± standard deviation (mean ± SD). Independent sample t-test or paired t-test was used for comparisons between two groups, and one-way ANOVA and *post hoc* multiple comparisons were used for comparisons among multiple groups. Counting data were analyzed using the χ² test or Fisher’s exact test. Correlation analysis was conducted using Pearson or Spearman methods. Survival analysis was performed using the Kaplan-Meier method and the log-rank test. Statistical analysis was performed in GraphPad Prism 10.1.2 or R Studio 4.3.1. A two-sided *P* < 0.05 was considered statistically significant.

## 3 Results

### 3.1 Bioinformatics screening and data mining

**Figure 1:**
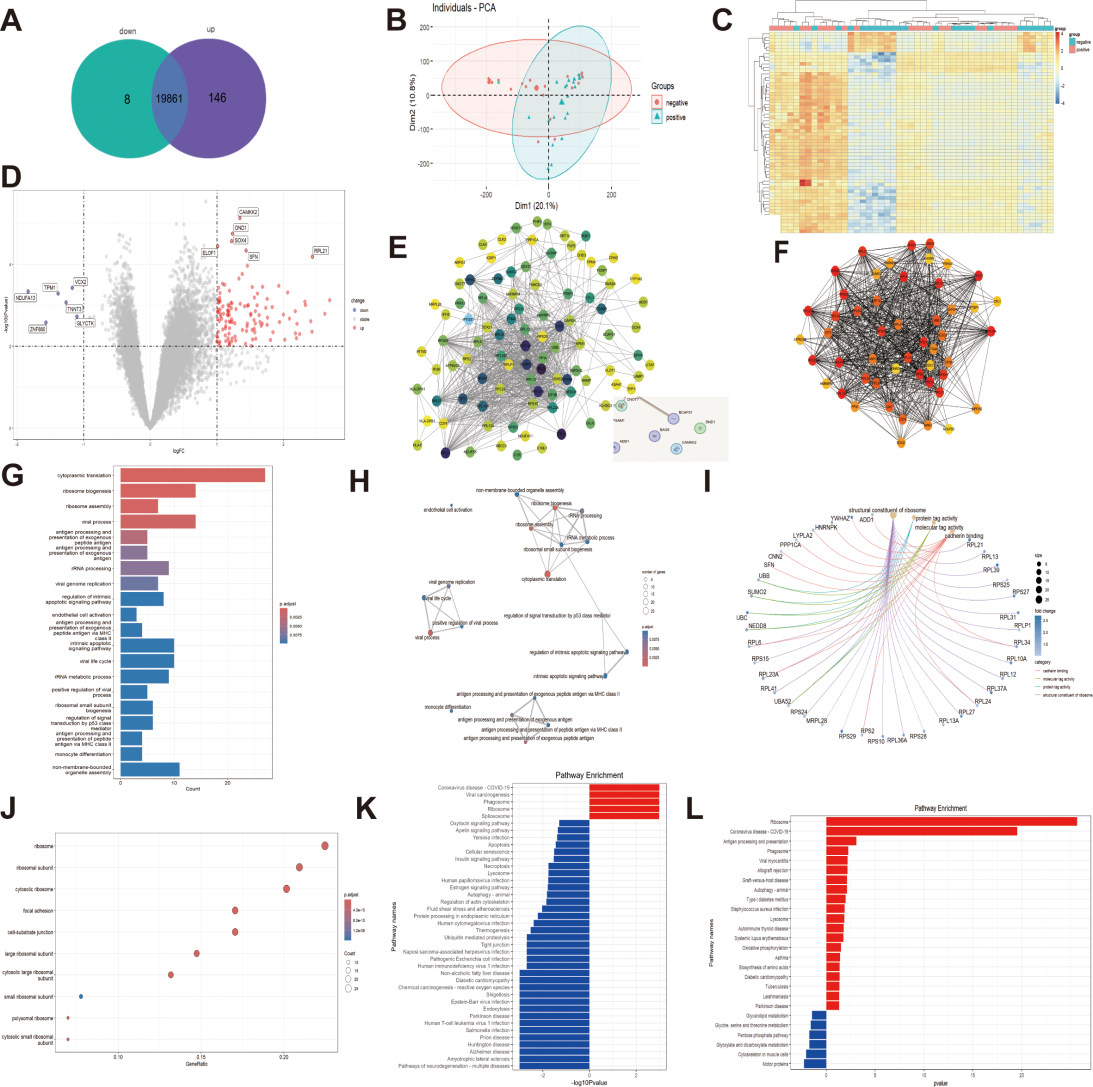
Bioinformatics screening of differential genes, SOX4 source and enrichment analysis **(A)** Venn diagram showing the screening of aberrant up- and down-regulated genes in GSE201777; **(B)** Principal component analysis (PCA) plot showing the differences in lymph node metastasis status between the two groups of samples; **(C)** Differential expression heatmap of up- and down-regulated genes; **(D)** Volcano plot labeling the candidate genes with significant differences; **(E)** PPI protein interactions network showing the potential interactions genes of SOX4; **(F)** MCC network map screening the top 50 up-regulated genes. **(G, H)** Bar and line graphs showing biological process (BP)-related enrichment analysis in GO; **(I, J)** Arc and dot plots showing molecular function (MF)-related pathway enrichment in GO; **(K, L)** Bar graphs showing KEGG and GSEA enrichment analysis.

After conducting differential expression analysis on the GSE201777 dataset, 146 up-regulated genes and 8 down-regulated genes were screened out (**Figure 1A**). After excluding non-tumor samples, principal component analysis (PCA) and differential expression heatmaps were performed on 47 LSCC tissue samples grouped by the presence or absence of lymph node metastasis, showing significant separation in the expression profiles of the two groups of samples (**Figures 1B, C**). Taking logFC > 1 and -log10 (P value) ≥ 2 as the screening criteria for up-regulated genes, and logFC < -1 and -log10 (P value) ≥ 2 as the screening criteria for down-regulated genes, all genes (including 146 up-regulated genes) were screened. Volcano plots were made for the eight down-regulated genes, and the three candidate genes with the greatest and most significant multiple changes among the up-regulated genes, SOX4, CAMKK2 and DND1, were screened out as the targets for subsequent research (**Figure 1D**). The differentially expressed genes in the volcano map were imported into STRING to construct the PPI network, and the results suggested that SOX4 and SMAD4 might interact (**Figure 1E**). In Cytoscape, the top 50 upregulated genes with the highest MCC values were sorted by maximum group centrality (MCC) (**Figure 1F**).

Pathway enrichment analysis was conducted on SOX4, CAMKK2 and DND1. GO biological process analysis suggests that SOX4-related pathways are enriched in signal transduction regulation dominated by p53-like mediators (**Figures 1G, H**); However, no significant items of co-enrichment of the three genes were observed in the top 5/20 items of GO molecular functions (**Figures 1I, J**). KEGG and GSEA enrichment indicated that the CAMKK2-related pathways included autophagy and oxytocin signaling pathways (appearing in the top 30 pathways with more significant P-values, **Figures 1K, L**). In conclusion, the bioinformatics results suggest that SOX4 and CAMKK2 have suspicious abnormal expressions and related pathways in the sample groups with and without lymph node metastasis, while DND1 mainly shows expression differences.

### 3.2 Expression verification of SOX4, CAMKK2 and DND1 in cells and tissues

**Figure 2:**
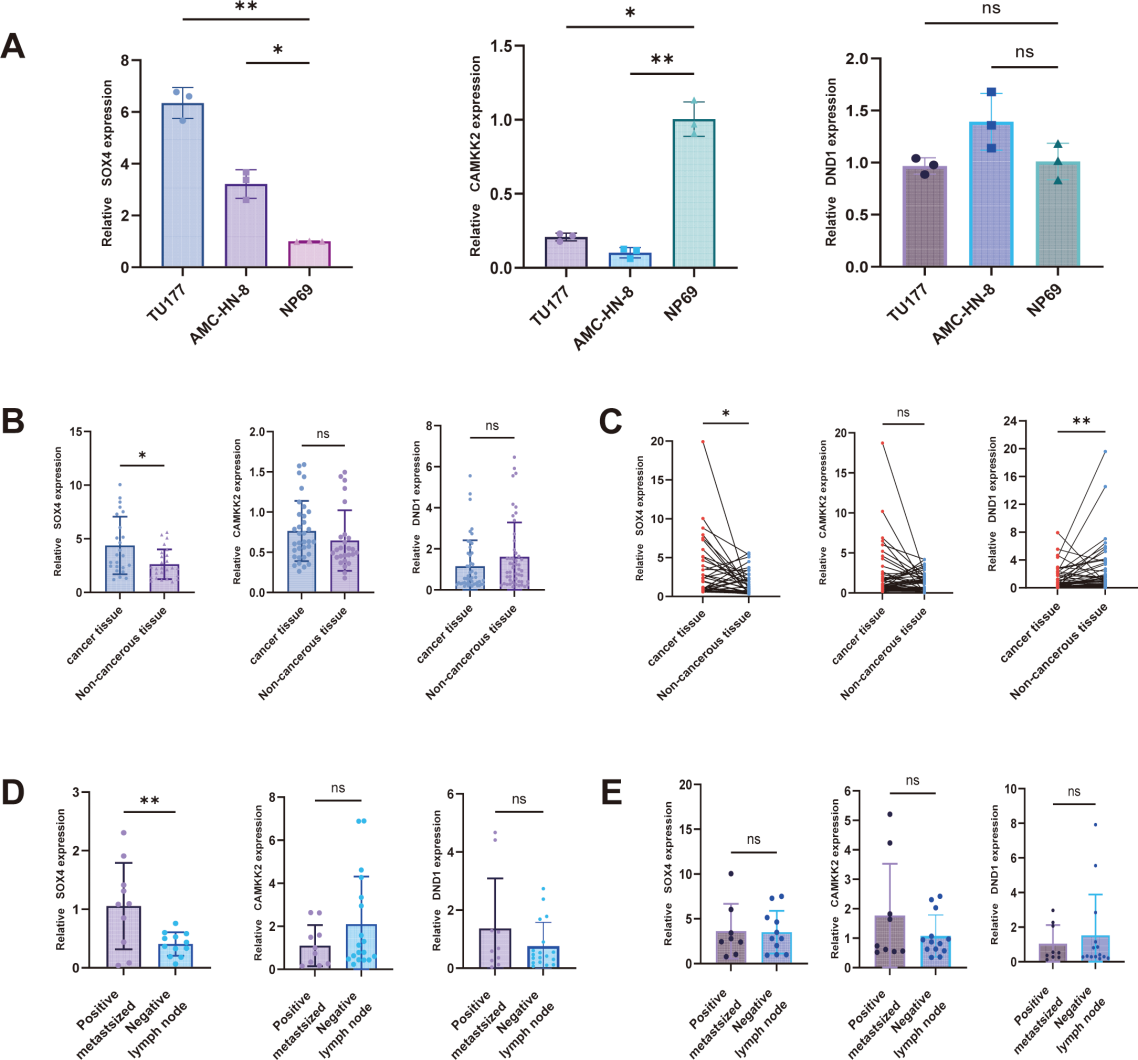
Expression levels of SOX4, CAMKK2 and DND1 in cells and tissues **(A)** Bar graph showing the expression levels of SOX4, DND1 and CAMKK2 in three cell types; **(B)** Bar graph showing the expression levels of the three genes in laryngeal cancer tissues versus paired non-cancerous tissues; **(C)** Pairwise connectivity plots showing the expression of SOX4, CAMKK2 and DND1 in cancerous tissues versus non-cancerous tissues; **(D, E)** Bar graph showing the expression of SOX4, CAMKK2 and DND1 in the same T1/T2 and same T3/T4 stage and lymph node metastasis-related cancer tissues. DND1 expression in cancer tissues with the same T1/T2 and the same T3/T4 stage and lymph node metastasis-related cancer tissues. *P < 0.05, ** *P* < 0.01.

**Figure 3:**
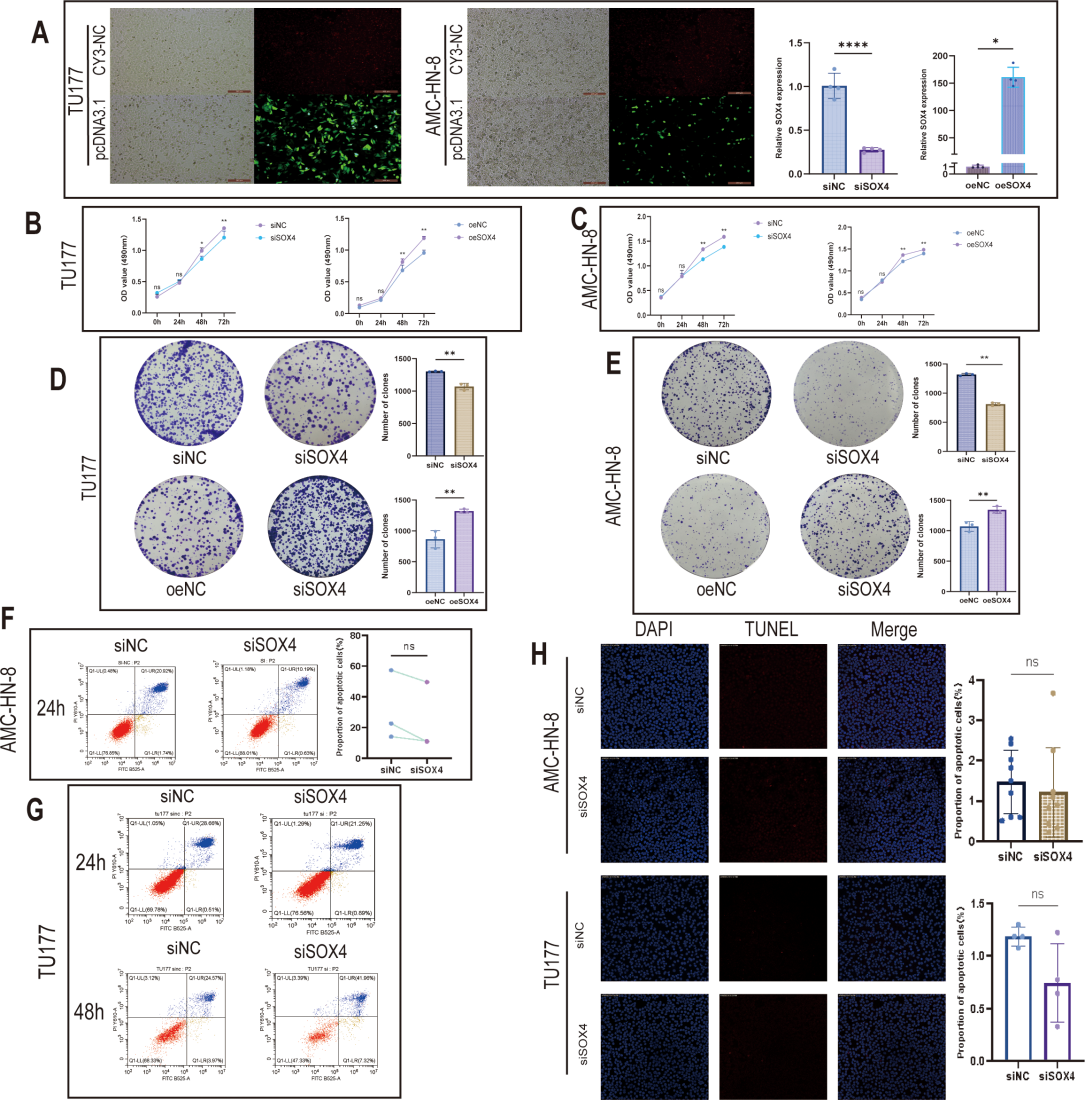
Construction of SOX4 knockdown and overexpression cell lines and effects on cell proliferation and apoptosis **(A)** Bar graph showing the validation of SOX4 knockdown and overexpression efficiencies in TU177 and AMC-HN-8; **(B, C)** Folding line graphs showing the change of absorbance of laryngeal cancer cells from 0-72h in MTS assay; **(D, E)** Results of clone formation assay showing the change of laryngeal cancer cell proliferation after knockdown and overexpression of SOX4; **(F–H)** Flow cytometry and TUNEL assay showing the knockdown of SOX4 on cell apoptosis. *P < 0.05, **P < 0.01, ****P < 0.0001.

At the cell line level, qRT-PCR showed that SOX4 was upregulated in tumor cell lines (TU177, AMC-HN-8), CAMKK2 expression was downregulated, and there was no significant difference in DND1 (**Figure 2A**). In the clinical samples, the qRT-PCR verification results for 58 pairs of tumor/adjacent non-tumor tissues were consistent with the cell lines (**Figure 2B**). Further repeated testing with 55 pairs of samples showed that SOX4 was significantly elevated in tumor tissues, CAMKK2 expression was relatively decreased, and the expression trend of DND1 was consistent with the above (**Figure 2C**). In the group comparison based on TNM staging (T1/T2 group versus T3/T4 group) and distinguishing whether lymph node metastasis occurred, only SOX4 was significantly elevated in the lymph node metastasis positive group (**Figures 2D, E**). Based on the above evidence, we selected SOX4 as the main target gene for the subsequent functional study and constructed the AMC-HN-8 and TU177 cell lines transfected with siSOX4 and oeSOX4. Experimental verification showed that both knockdown and overexpression were successful (**Figure 3A**).

### 3.3 The effect of SOX4 on cell proliferation

By MTS proliferation detection, the OD values of the SOX4 knockdown group (AMC-HN-8, TU177) were significantly decreased compared with the control group (*P* < 0.05), while the OD values of the SOX4 overexpression group were significantly upregulated (*P* < 0.01) (**Figures 3B, C**). The clone formation experiment showed that overexpression of SOX4 could significantly increase the clone numbers of TU177 and AMC-HN-8 (all *P* < 0.01), while knockdown of SOX4 significantly reduced the clone formation ability of the two cell lines (all *P* < 0.01) (**Figures 3D, E**). The above results indicate that SOX4 can promote the proliferation and clone formation ability of LSCC cells *in vitro* and has a cancer-promoting effect.

### 3.4 The effect of SOX4 knockdown on apoptosis

After evaluating the apoptosis rate by flow cytometry and TUNEL method, it was found that compared with the control group, there was no significant difference in the early/late apoptosis ratio between AMC-HN-8 with knocked SOX4 and TU177 at 24 h (and 48 h of TU177) (**Figures 3F–H**). The TUNEL staining results also showed no significant changes. It can be seen from this that SOX4 knockdown did not significantly affect the apoptosis levels of the two LSCC cell lines under the conditions of this experiment.

### 3.5 The effect of SOX4 on cell migration ability (scratch and Transwell migration assay)

**Figure 4:**
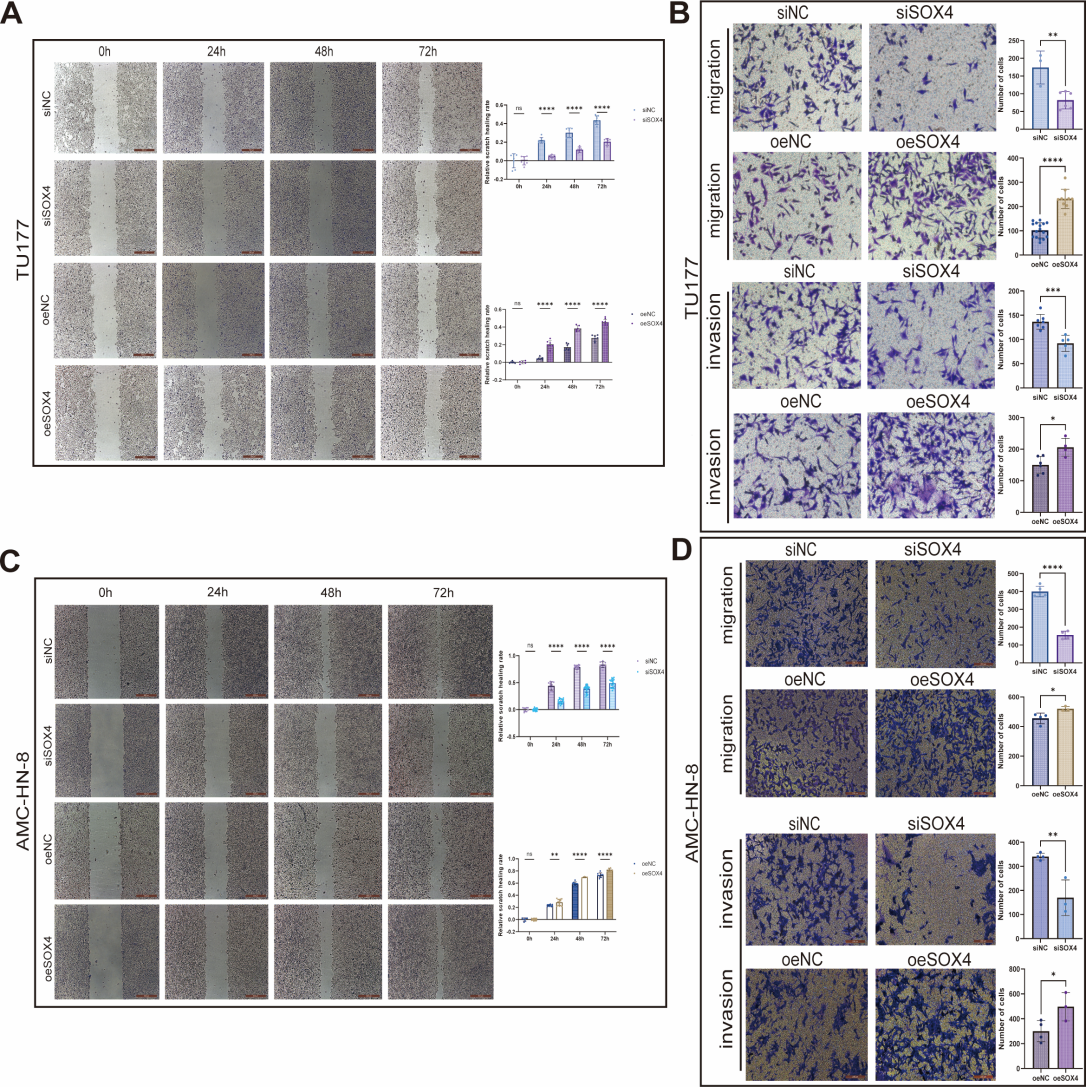
Modulation of migration and invasion ability of laryngeal squamous carcinoma cells by SOX4 **(A)** Scratch healing assay showing the 0-72h TU177 scratch healing rate after knockdown and overexpression of SOX4; **(B)** Transwell assay showing that knockdown of SOX4 significantly decreased the number of TU177 perforating cells and overexpression significantly increased the number of perforating cells; **(C)** Scratch healing assay showing the 0-72h AMC-HN-8 scratch healing rate after knockdown and overexpression of SOX4; **(D)** Transwell assay showing that knockdown of SOX4 significantly decreased the number of AMC-HN-8 perforated cells and overexpression significantly increased the number of perforated cells. *p < 0.05, **p < 0.01, ***p < 0.001, **** *p* < 0.0001.

The scratch healing experiment showed that the scratch healing rate of AMC-HN-8 or TU177 cells knocked down with SOX4 was significantly decreased (*P* < 0.001), while the healing rate of the SOX4 overexpression group was significantly increased (*P* < 0.01) (**Figures 4A, C**). The results of the Transwell migration assay were consistent with those of the scratch assay: knockdown of SOX4 significantly reduced the number of cells passing through the basement membrane of AMC-HN-8 (*P* < 0.001) and TU177 (*P* < 0.01). Overexpression of SOX4 significantly increased the migration numbers of the two cell lines (AMC-HN-8 *P* < 0.05; TU177 *P* < 0.001) (**Figures 4B, D**). In conclusion, SOX4 can significantly promote the migration ability of LSCC cells.

### 3.6 The effect of SOX4 on cell invasion cbility (Transwell invasion assay)

In the Transwell invasion assay coated with Matrigel, overexpression of SOX4 significantly increased the number of AMC-HN-8 and TU177 cells passing through the basement membrane (*P* < 0.05); Conversely, knockdown of SOX4 significantly reduced the number of invasive cells in the two cell lines (AMC-HN-8 *P* < 0.01; TU177 *P* < 0.005) (**Figures 4B, D**). These data further support the role of SOX4 in enhancing cell invasiveness in LSCC.

### 3.7 Transcriptomic screening of downstream target genes of SOX4

Transcriptome sequencing was performed on siSOX4 and SINC-treated TU177 cells, and the results showed a significant separation between the two groups (**Supplementary Figure S2A**). Compared with the control group, the siSOX4 group had a total of 141 up-regulated genes and 137 down-regulated genes. According to the screening criteria of log2FC > 1 and -log10 (P value) > 90, the candidate genes included PTBP2, IFI6, IFI27 and SOD1 (**Supplementary Figures S2B, C**). GO enrichment suggests that PTBP2, IFI6 and IFI27 are involved in the formation process, stress response and biological regulation. At the cellular component level, IFI6 and IFI27 were associated with membranes (*P* < 0.05) (**Supplementary Figure S2D**). Disease ontology (DO) enrichment analysis revealed that IFI27 and SOD1 were involved in the tumorigenesis pathway related to proliferation, while PTBP2 was associated with SOD1 in the hepatobiliary system tumor pathway (*P* < 0.05) (**Supplementary Figure S2E**). Subsequently, the expression of the above candidate genes was verified by qRT-PCR in the cell lines. The results showed that only the expression change of PTBP2 was consistent with the transcriptome sequencing: the expression of PTBP2 decreased with SOX4 knockdown in both LSCC cell lines (**Supplementary Figure S2F**). Based on this, PTBP2 was selected as the subsequent downstream research object.

### 3.8 Bioinformatics correlation between SOX4 and PTBP2 in tumor immunity and drug sensitivity

#### 3.8.1 Immune cell infiltration is associated with survival

**Figure 5:**
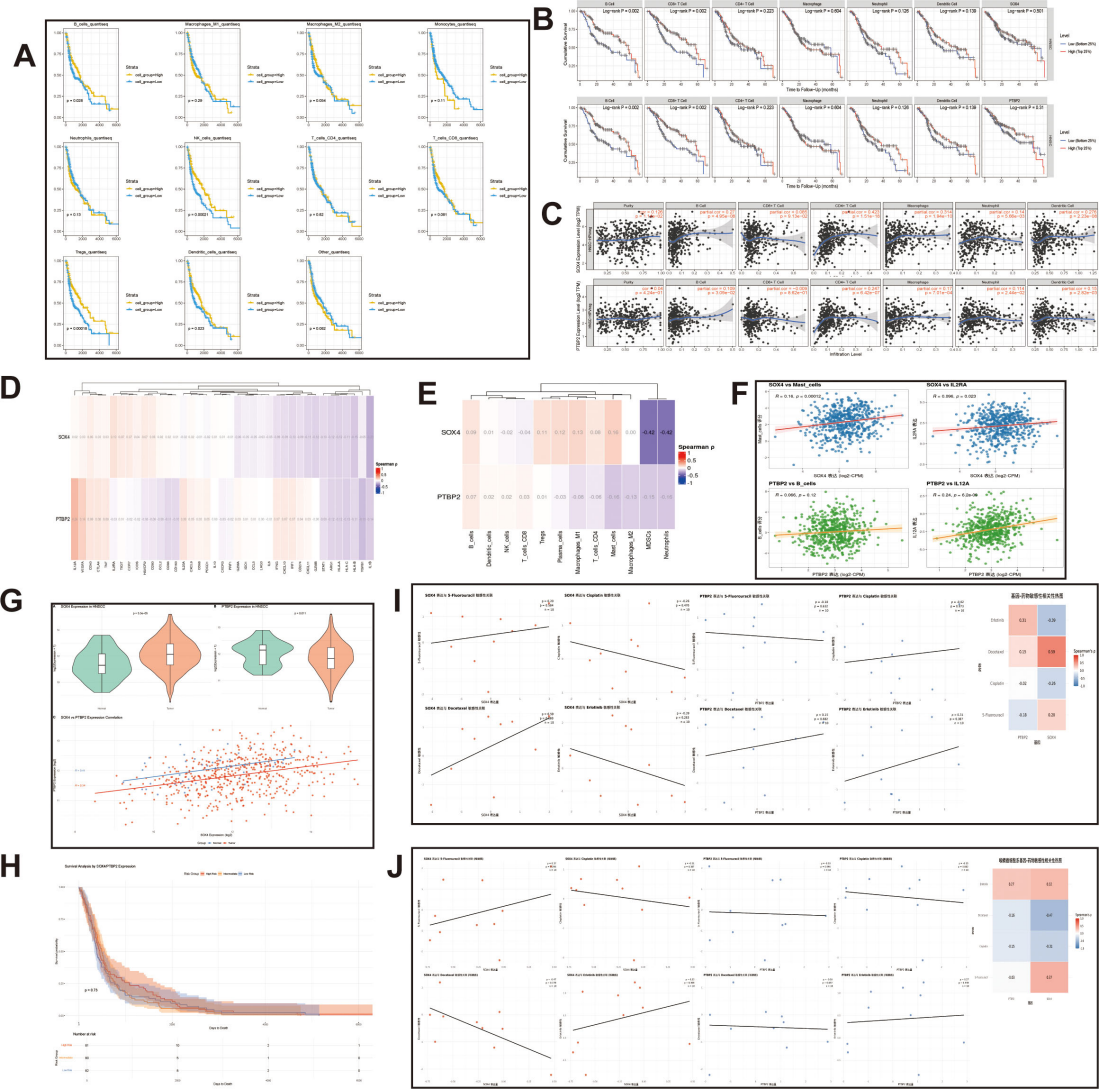
Relationship of SOX4 and PTBP2 with the immune microenvironment, patient prognosis and drug sensitivity **(A)** Curve plot analysis of TCGA showing the correlation between immune cell levels and survival in laryngeal cancer tissues; **(B)** Curve plot analysis of TIMER showing the correlation between immune cell levels and survival in laryngeal cancer tissues; **(C)** Scatterplot showing the correlation between SOX4 and PTBP2 expression and six immune cells; **(D, E)** Correlation of SOX4 and PTBP2 with immune factors and immune cells heatmap; **(F)** Scatterplot fitting showed that SOX4 was significantly positively correlated with mast cells and IL2RA, and PTBP2 was positively correlated with B cells and IL12A; **(G)** Violin plot showed that the expression of the two genes was significantly higher in tumor tissues than in normal tissues and the scatterplot showed a strong positive correlation between the two genes; **(H)** Risk-grouped survival curves suggesting that SOX4 and PTBP2 may synergistically promote tumor progression; **(I, J)** Scatter plot showing the correlation of SOX4 and PTBP2 expression with chemotherapeutic agents.

The relationship between immune cell expression and survival in head and neck squamous cell carcinoma (HNSC) was analyzed using the TCGA and TIMER2.0 databases. The results showed that elevated levels of B cells, NK cells, regulatory T cells and dendritic cells were associated with prolonged survival of patients (*P* < 0.05, **Figure 5A**). In TIMER 2.0, when the infiltration of B cells and CD8^+^T cells increased, the 5-year survival rate of patients significantly improved (*P* < 0.001). Although the high expression of SOX4 and PTBP2 within a 5-year period did not show a significant survival difference, the survival rate of the high-expression group decreased over time (more than 5 years), suggesting that the two genes have potential carcinogenic effects (**Figure 5B**).

#### 3.8.2 Correlation between SOX4/PTBP2 and immune cells and immune factors

In the correlation analysis of Timer 2.0, the expressions of B cells, CD4^+^T cells, macrophages, neutrophils and dendritic cells were all significantly positively correlated with SOX4 and PTBP2 (*P* < 0.05) (**Figure 5C**). The immune correlation heatmaps (**Figures 5D, E**) and scatter fitting analysis (**Figure 5F**) of TCGA data show that SOX4 has a strong correlation with mast cells (*P* < 0.001) and IL2RA (*P* < 0.05). PTBP2 has the strongest correlation with B cells and IL12A (*P* < 0.001).

#### 3.8.3 Analysis of tissue expression distribution and co-expression

In the TCGA HNSC dataset, the expressions of SOX4 and PTBP2 were more concentrated in tumor tissues (compared with normal tissues, *P* < 0.05), and the two genes were significantly positively correlated in cancer tissues (**Figure 5G**). The risk-grouped survival curves based on two genes show that the abnormality of a single gene has no significant impact on survival risk, suggesting that SOX4 and PTBP2 may affect tumor progression through a synergistic effect (**Figure 5H**).

#### 3.8.4 Drug sensitivity analysis

The fitting analysis of drug sensitivity using DepMap data shows that among several anti-tumor drugs, SOX4 has a relatively high correlation with taxanes (such as docetaxel). The sensitivity correlation between PTBP2 and Erlotinib is relatively strong (**Figure 5I**). In the drug sensitivity analysis for LSCC, SOX4 was highly sensitive to 5-fluorouracil, while PTBP2 was highly sensitive to Erlotinib (**Figure 5J**). Although not all of these differences were statistically significant, they suggested that SOX4/PTBP2 might be associated with specific drug responses.

### 3.9 Verification of PTBP2 expression in cells

In the cell models of knockdown and overexpression of SOX4 (TU177, AMC-HN-8), qRT-PCR verification showed that: Knockdown of SOX4 leads to down-regulation of PTBP2 expression, while overexpression of SOX4 can up-regulate PTBP2 (**Supplementary Figures S3A, B**), suggesting that the expression trend of PTBP2 is consistent with that of SOX4. Subsequently, the siPTBP2 and oePTBP2 transfection systems were constructed and verified. The results showed that both knockdown and overexpression of PTBP2 were successful (*P* < 0.0001), which could be used for subsequent functional detection (**Supplementary Figure S3C**).

### 3.10 The effect of PTBP2 on cell proliferation (colony formation experiment)

The clone formation experiment indicated that overexpression of PTBP2 significantly increased the number of clones of TU177 (*P* < 0.001) and AMC-HN-8 (*P* < 0.05). Knockdown of PTBP2 significantly reduced the clonal formation ability of the two cell lines (TU177, AMC-HN-8, both *P* < 0.001) (**Supplementary Figures S3D, E**). The results suggest that PTBP2 also has the function of promoting the proliferation of LSCC cells *in vitro*.

### 3.11 The influence of PTBP2 on migration and invasion

The scratch and Transwell migration/invasion experiments showed that PTBP2 knockdown significantly inhibited the migration and invasion of AMC-HN-8 and TU177 (the scratch healing rate and the number of Transwell migration/invasion cells were all *P* < 0.001); PTBP2 overexpression significantly promoted the migration and invasion abilities of the two cell lines (the scratch healing rate and the number of Transwell migration/invasion cells reached *P* < 0.01~*P* < in multiple comparisons). 0.0001) (**Supplementary Figures S3F–I**). In summary, PTBP2 promotes the migration and invasion of LSCC.

### 3.12 Rescue experiment: verification of the synergistic effect of PTBP2 and SOX4

#### 3.12.1 Experimental design and cell line construction

Cell lines simultaneously containing the PTBP2 mutant and knockdown SOX4, as well as cell lines knockdown PTBP2 and overexpressing SOX4, were constructed and functionally verified in TU177 and AMC-HN-8, respectively.

#### 3.12.2 Clone formation assay (proliferation)

**Figure 6:**
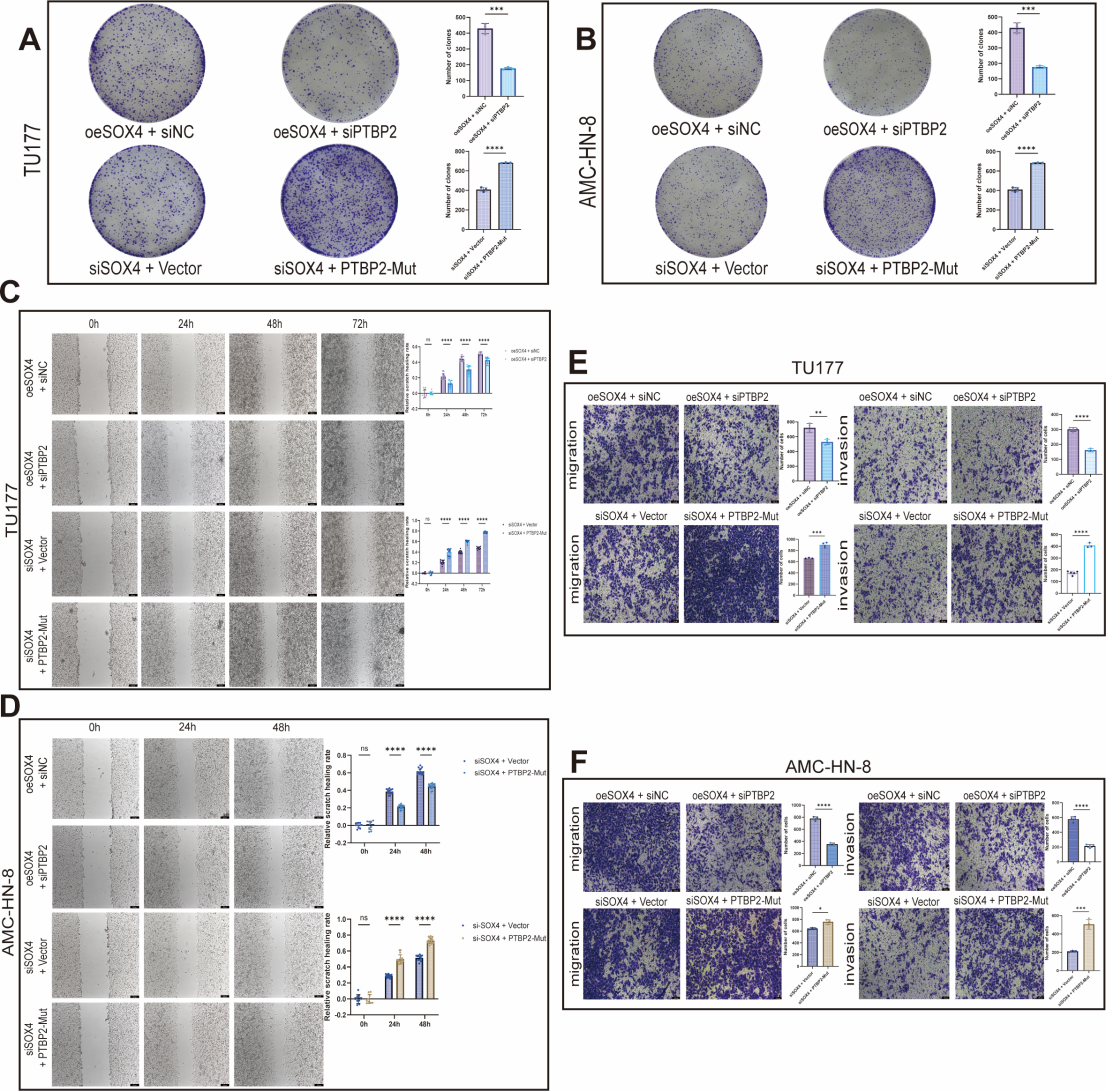
PTBP2 inversely regulates SOX4-regulated cellular phenotypes **(A, B)** Clone formation experiments show that PTBP2 alleviates proliferation inhibition caused by SOX4 knockdown, and knockdown of PTBP2 inhibits proliferation enhancement induced by SOX4 overexpression; **(C, D)** Scratch experiments show that PTBP2 knockdown inhibits migration enhancement induced by SOX4 overexpression, and PTBP2 partially restores migratory ability after SOX4 knockdown; **(E, F)** Transwell experiments validate the critical synergistic role of PTBP2 in SOX4-regulated migration and invasion. *p < 0.05, **p < 0.01, ***p < 0.001, ****P < 0.0001.

The SOX4 knockdown group with mutant PTBP2 significantly restored the clone formation ability compared with the SOX4 knockdown group only (TU177, AMC-HN-8, both *P* < 0.0001); However, under the conditions of knocking down PTBP2 and overexpressing SOX4, the clonal increase effect induced by overexpression SOX4 was partially offset (TU177 *P* < 0.001; AMC-HN-8 *P* < 0.01) (**Figures 6A, B**). These data indicate that PTBP2 can partially alleviate the proliferation inhibitory effect caused by the knockdown of SOX4, and the two have a synergistic relationship in proliferation regulation.

#### 3.12.3 Scratches and transwell migration (migration)

In the migration experiment, the scratch healing rate and Transwell migration number of cells that knocked down PTBP2 and overexpressed SOX4 were significantly lower than those of the control group that only overexpressed SOX4 (*P* < 0.0001 to *P* < 0.001). It is indicated that knocking down PTBP2 can inhibit the migration enhancement induced by SOX4. Conversely, the PTBP2 mutant can partially restore its migration ability in the context of knockdown of SOX4 (**Figures 6C–F**). This proves that PTBP2 and SOX4 have functional interactions in migration regulation.

#### 3.12.4 Transwell invasion (invasion)

The results of the invasion experiment are consistent with the trends of migration and proliferation: The invasion ability of the SOX4 knockdown group with mutant PTBP2 was higher than that of the SOX4 knockdown group only (both TU177 and AMC-HN-8 were significant). However, knocking down PTBP2 could significantly weaken the enhanced invasion caused by overexpression of SOX4 (*P* values mostly ranged from < 0.0001 to < 0.001) (**Figures 6E, F**). In conclusion, PTBP2 plays a significant downstream or auxiliary role in the SOX4-mediated LSCC oncogenic phenotype, and the two work in synergy to promote cell proliferation, migration and invasion.

### 3.13 *In vivo* tumor-bearing experiments: effects of SOX4 and Erlotinib on tumor growth and lymph node metastasis

#### 3.13.1 Animal models and grouping

**Figure 7:**
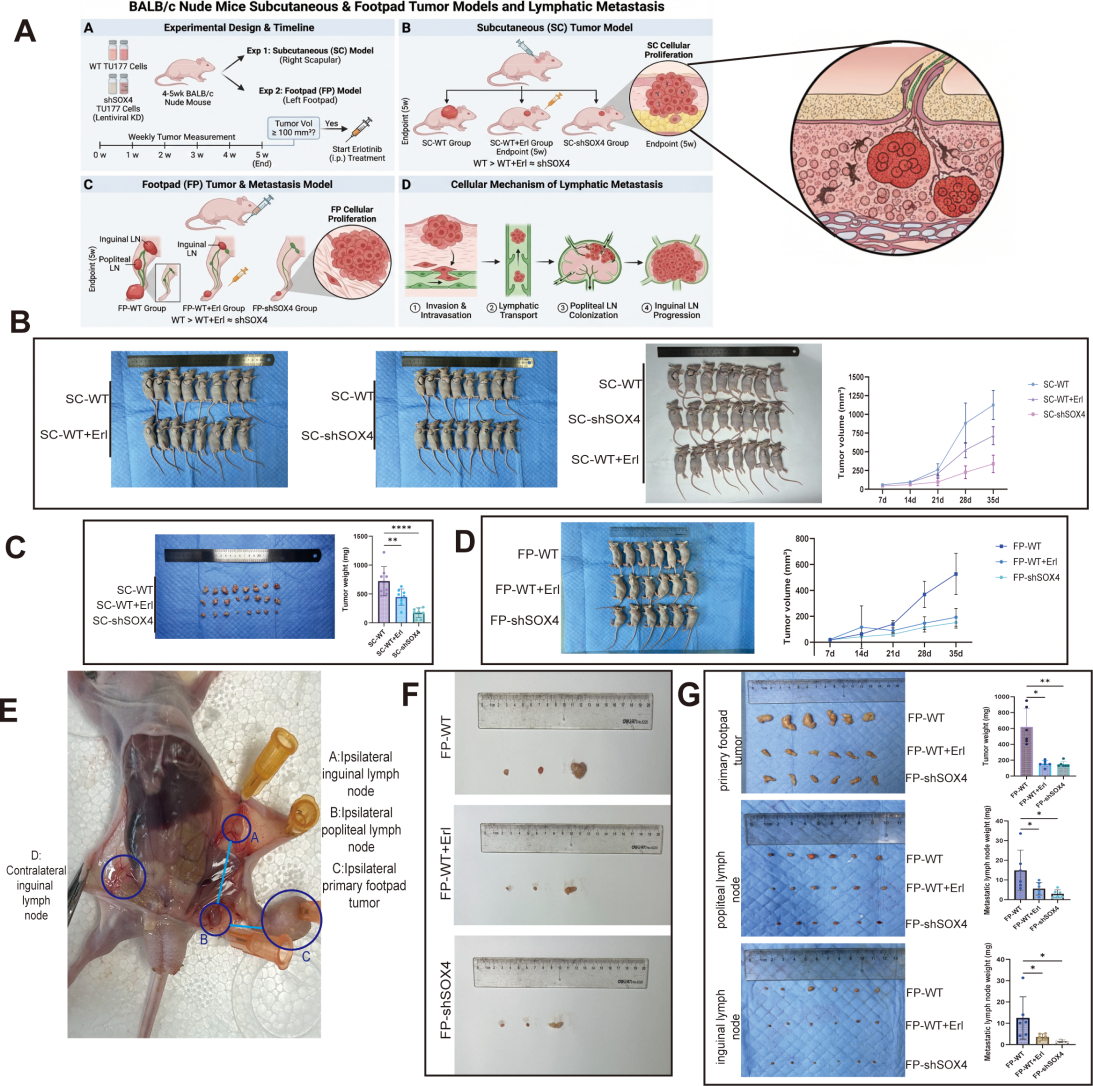
*In vivo* validation of SOX4 on proliferation, metastasis and drug response in laryngeal squamous carcinoma **(A)** Schematic diagram of the nude mouse loaded tumor experimental model; **(B)** Curve showing subcutaneous tumor growth showing that knockdown of SOX4 or Erlotinib inhibited tumor growth; **(C)** Bar graph statistics of subcutaneous tumor weight; **(D)** Tumor growth curve of the footpads; **(E)** Lymphatic metastatic pathway schematic; **(F)** Comparison of the volume of footpad-popliteal-femoral-inguinal lymph node metastasis system; **(G)** Statistics of the weight of each tissue. Showing that knockdown of either SOX4 or Erlotinib significantly inhibited tumor and lymph node metastasis. *p < 0.05, **p < 0.01, ****P < 0.0001.

A subcutaneous and foot pad injection xenograft model was established (subcutaneous right scapular and left foot pad), with a total of 24 mice subcutaneous right scapular and 21 mice on the left foot pad. The groups included wild-type control, Erlotinib administration group and SOX4 knockdown group (**Figure 7A**).

#### 3.13.2 Effects on tumor growth *in vivo*

The growth curves of subcutaneous and foot pad tumors showed that the tumor growth rates in the Erlotinib administration group and the SOX4 knockdown group were significantly lower than those in the control group (**Figure 7B**). At 28-day sampling, the average weight of subcutaneous tumors in the dosing group and the SOX4 knockdown group was significantly smaller than that in the control group (*P* < 0.01 in the Erlotinib group; *P* < 0.0001 in the SOX4 knockdown group) (**Figure 7C**), suggesting that both could inhibit the proliferation of LSCC *in vivo*.

#### 3.13.3 Impact on lymph node metastasis

In the foot pad model, both Erlotinib and SOX4 knockdown significantly slowed down the growth of *in situ* tumors in the foot pad (**Figure 7D**). At 28 days, the popliteal fossa and inguinal lymph nodes on the affected side were significantly enlarged. However, in the Erlotinib group and the SOX4 knockdown group, the volumes of the foot pad *in situ* tumor, popliteal fossa and inguinal lymph nodes were all smaller than those in the control group (**Figures 7E, F**). Specific comparisons showed that the weight of foot pad tumors and popliteal lymph nodes in the Erlotinib group and the SOX4 knockdown group was significantly lower than that in the control group (some *P* values < 0.05 to < 0.0001, see **Figure 7G**). These *in vivo* data confirm that both Erlotinib treatment and SOX4 knockdown can inhibit the *in vivo* proliferation of LSCC and weaken its lymph node metastasis ability.

## 4 Discussion

This study systematically elucidates the oncogenic role of the transcription factor SOX4 in laryngeal squamous cell carcinoma (LSCC) and clarifies the critical function of its downstream effector PTBP2 in SOX4-mediated tumor progression. Moving beyond a simple analysis of expression correlations, we expanded SOX4 research to multi-level functional and mechanistic validation both *in vitro* and *in vivo*, demonstrating that SOX4 is closely associated with lymph node metastasis at an early stage of LSCC development. Our results show that SOX4 is significantly overexpressed in LSCC tissues and cell lines and is positively correlated with lymph node metastasis. Functional assays further confirm that SOX4 markedly promotes LSCC cell proliferation, migration, and invasion, while exerting relatively limited effects on apoptosis. Through transcriptome sequencing combined with functional screening, we identified PTBP2 for the first time as a key downstream effector of SOX4 in LSCC. Rescue and reversal experiments demonstrated that alterations in PTBP2 expression can partially reverse the malignant phenotypes mediated by SOX4. *In vivo* animal experiments further confirmed that either SOX4 knockdown or Erlotinib treatment significantly suppresses tumor growth and reduces lymph node metastasis.

Although SOX4 knockdown reduced migration and invasion in our assays, the apoptosis data showed no significant change under the same experimental conditions, and the motility assays were conducted in a short time window after transfection. Therefore, the observed phenotype is unlikely to be explained solely by apoptosis-related cell loss; however, we acknowledge that future studies using proliferation-blocking controls or real-time motility systems would further exclude proliferation-dependent confounding.

LSCC is one of the most common malignant tumors of the head and neck. Owing to the lack of specific early clinical symptoms, most patients are diagnosed at advanced or late stages, often accompanied by local invasion and cervical lymph node metastasis, resulting in an unfavorable overall five-year survival rate (Zhang et al., 2023) (12). Cervical lymph node metastasis is considered a core determinant in prognostic evaluation and treatment decision-making for LSCC patients; however, conventional imaging and clinical examinations remain limited in their ability to detect micrometastatic lesions (Kim et al., 2013) (13). Therefore, systematically elucidating the key molecular networks driving LSCC initiation, progression, and metastasis is of great significance for identifying novel diagnostic biomarkers, accurately assessing metastatic risk, and formulating individualized therapeutic strategies.

In recent years, the roles of SOX family transcription factors in precise oncogenic regulatory networks have become increasingly evident. Among them, SOX4 has attracted considerable attention due to its critical functions in embryonic development and cell fate determination (Yu et al., 2023; Lu et al., 2017) (14, 15). Previous studies have shown that SOX4 is aberrantly overexpressed in multiple solid tumors, including esophageal squamous cell carcinoma, breast cancer, and lung cancer, where it promotes tumor cell proliferation, epithelial–mesenchymal transition (EMT), invasion, migration, and therapeutic resistance by regulating downstream gene networks (Chen et al., 2021; Nan et al., 2022; Wang et al., 2026) (16–18), and in certain tumor types participates in the regulation of apoptosis (Long et al., 2024; Chen et al., 2021) (20, 21). Although aberrant SOX4 expression has been reported in head and neck cancers (Jang et al., 2025) (19), most studies are limited to expression differences and lack systematic elucidation of its functional roles and molecular mechanisms. Through multi-level experimental approaches, the present study clearly demonstrates the oncogenic function of SOX4 in LSCC and, for the first time, directly links it to the clinically critical event of lymph node metastasis, thereby providing more robust evidence for the biological significance of SOX4 in LSCC progression.

From a mechanistic perspective, the importance of RNA-binding proteins (RBPs) in post-transcriptional regulation of cancer has gained increasing recognition (Cai et al., 2021; Criscuolo et al., 2021) (27, 28). Polypyrimidine tract-binding protein 2 (PTBP2), a member of the PTBP family, is mainly involved in alternative splicing, mRNA stability, and translational regulation (Courraud et al., 2025) (29). Accumulating evidence indicates that PTBP2 is aberrantly expressed in various cancers and is closely associated with enhanced tumor cell proliferation, metabolic reprogramming, and invasive and metastatic capacities (Chen et al., 2022; Li et al., 2025) (30, 31). However, the functional role of PTBP2 in LSCC and its upstream regulatory mechanisms have not been previously clarified. In this study, we demonstrate for the first time that PTBP2 is a functional downstream target of SOX4 in LSCC, and through systematic knockdown, overexpression, and rescue experiments, we confirm their coordinated relationship at both the expression and biological function levels. We speculate that SOX4 may promote PTBP2 expression to remodel the post-transcriptional regulatory network of tumor cells, thereby affecting genes related to the cell cycle, cell adhesion, and extracellular matrix degradation, ultimately facilitating tumor cell proliferation and invasion (Bao et al., 2024) (22).

It should be noted that although this study functionally validates the existence of the SOX4–PTBP2 axis in LSCC, whether SOX4 exerts transcriptional regulation by directly binding to the promoter or enhancer regions of PTBP2, or instead regulates PTBP2 indirectly through intermediary factors, remains to be determined. Future studies may employ ChIP-qPCR or ChIP-seq to identify SOX4 binding sites within PTBP2 regulatory regions, and integrate RIP-seq, CLIP-seq, or differential splicing analyses to systematically delineate downstream splicing targets of PTBP2 and their enriched functional pathways, thereby further refining the regulatory model of the SOX4–PTBP2 axis at the molecular level.

Another limitation of the present study is that tissue-level protein validation by immunohistochemistry was not performed for SOX4, CAMKK2, DND1, or PTBP2. The paired fresh tissue specimens were limited and were fully consumed for qRT-PCR analysis, which prevented additional immunohistochemical validation of protein expression in the same cohort. Future studies using an independent sample set should further confirm the tissue-level protein expression pattern/spatial distribution of SOX4 and its related molecules.

In addition, by integrating molecular mechanism exploration with analyses of the tumor immune microenvironment and drug sensitivity, this study preliminarily suggests that the SOX4–PTBP2 axis may play an important role in tumor immune regulation and therapeutic response. Analyses of TCGA and TIMER datasets reveal that SOX4 and PTBP2 expression levels are significantly correlated with infiltration of multiple immune cell types, including B cells, CD4^+^ T cells, and macrophages (Rossi et al., 2025; Ogbodo et al., 2024) (25, 26), and that their high expression is associated with a trend toward poor prognosis, suggesting that this axis may indirectly promote tumor progression by remodeling the immune microenvironment. At the pharmacological level, DepMap data analyses indicate a potential association between SOX4, PTBP2, and Erlotinib (Fang et al., 2025^)^ (23). Consistently, our *in vivo* experiments show that Erlotinib partially suppresses tumor growth and lymph node metastasis (Arrigo et al., 2023) (24), implying that combined targeting of the SOX4–PTBP2 axis and the EGFR signaling pathway may be theoretically feasible. Nevertheless, these database-based associations and animal experiment findings remain preliminary, and further validation through systematic *in vitro* dose–response assays, drug combination index analyses, and PDX/PDXO models is required.

From a clinical perspective, recent multicenter real-world data have highlighted substantial heterogeneity in treatment regimens and reinforced the importance of surgical nodal management in laryngeal and hypopharyngeal squamous cell carcinoma (Knopf et al., 2025; Ketterer et al., 2020) (32, 33). These findings are consistent with our observation that SOX4 is associated with lymph node metastasis, and they support the potential utility of the SOX4–PTBP2 axis as a molecular aid for risk stratification and nodal decision-making in LSCC.

Besides, from a translational clinical perspective, the SOX4–PTBP2 axis in LSCC possesses dual value as both a biomarker and a potential therapeutic target. On the one hand, incorporating SOX4 and PTBP2 into preoperative or postoperative risk assessment systems may help optimize cervical lymph node management strategies and adjuvant treatment decisions. On the other hand, the observation that SOX4 knockdown or Erlotinib treatment significantly inhibits tumor growth and reduces lymph node metastasis *in vivo* provides experimental support for combined molecular targeted therapy strategies. However, clinical application will require further validation in larger, multicenter clinical cohorts, together with in-depth mechanistic studies to clarify its druggability and safety.

Beyond tumor-cell intrinsic signaling, LSCC progression should also be viewed within a broader ecological and evolutionary framework involving the tumor microenvironment, stromal interactions, and therapeutic selection pressure. Recent studies have proposed cancer as a pathological ecosystem and emphasized the importance of ecological-evolutionary principles in understanding tumor initiation, progression, metastasis, and recurrence (Luo.2023) (34). Consistent with this perspective, post-genomic rethinking of cancer suggests that recurrence and metastasis cannot be fully explained by a single-gene or mutation-centered model alone (Luo.2025) (35). In the present study, our data support the SOX4–PTBP2 axis as a mechanistic driver of LSCC progression; however, this axis likely operates within a wider tumor ecosystem, which warrants future validation using spatial and single-cell approaches.

In summary, through an integrated approach encompassing bioinformatic screening, *in vitro* functional assays, and *in vivo* animal models, this study systematically elucidates the molecular mechanism by which SOX4 promotes tumor cell proliferation, migration, invasion, and lymph node metastasis in LSCC via upregulation of PTBP2. These findings not only deepen our understanding of the molecular basis underlying LSCC invasion and metastasis, but also, for the first time, propose a potential functional model of the SOX4–PTBP2 axis in lymphatic metastasis from a post-transcriptional regulatory perspective, providing new theoretical insights and research directions for precision stratification and targeted therapy in LSCC.

## 5 Conclusion

In conclusion, this study revealed the synergistic carcinogenic effect of the transcription factor SOX4 and its downstream effector molecule PTBP2 in laryngeal squamous cell carcinoma (LSCC). Both jointly promote the proliferation, migration, invasion and lymph node metastasis of LSCC cells, constituting a key functional regulatory axis. Our results suggest that the SOX4-PTBP2 axis not only drives the progression of LSCC at the molecular mechanism level but may also become a potential therapeutic target for LSCC in the future, providing new ideas for precise intervention.

## Data Availability

The datasets presented in this study can be found in online repositories. The names of the repository/repositories and accession number(s) can be found in the article/**Supplementary Material**.

## Supplementary material

Supplementary Figure 1 Statistical data, clinical staging and metastatic characteristics of laryngeal cancer in 2026 by World Health Organization (WHO) global cancer **(A)** Global prevalence of laryngeal cancer in China; **(B)** Incidence rate and distribution of laryngeal cancer among men and women in various regions of the world; **(C)** Incidence rate and mortality rate of laryngeal cancer among tumors in the whole body; **(D)** Clinical common laryngeal cancer subtypes; and **(E)** Clinical common metastatic sites of laryngeal cancer.

Supplementary Figure 2 SOX4 downstream target gene screening and validation **(A)** PCA plots of cellular transcriptome sequencing and differential gene Venn diagrams after knockdown of SOX4; **(B, C)** Volcano plots and heatmaps showing differential differentially differentiated genes with significantly up-regulated and down-regulated expression; **(D)** Bar graphs and bar graphs showing GO database annotation analyses and enrichment analyses of pathways, respectively; **(E)** Bar graphs and bar graphs showing DO database annotation analyses and enrichment analyses of pathways, respectively; **(F)** Bar graphs showing the cellular expression of PTBP2, IFI6, and IFI27 after knockdown of SOX4. **P* < 0.05, ** *P* < 0.01, *** *P* < 0.001.

Supplementary Figure 3 PTBP2 expression in laryngeal squamous carcinoma cells and its effect on cell proliferation, migration and invasion ability **(A)** Bar graph showing the expression level of PTBP2 in TU177 after knockdown/overexpression of SOX4; **(B)** Bar graph showing the expression level of PTBP2 in AMC-HN-8 after knockdown/overexpression of SOX4; **(C)** Bar graph showing the expression efficiency of PTBP2 after knockdown/overexpression of PTBP2 in TU177; **(D, E)** Results of the clone formation assay showing the proliferation changes in laryngeal cancer cells after knockdown/overexpression of SOX4; **(F, G)** Bar graph showing the cell scratch healing rate after knockdown and overexpression of PTBP2; **(H, I)** Transwell assay showing that PTBP2 regulates the migration and invasion ability of laryngeal cancer cells. **P* < 0.05, ** *P* < 0.01, *** *P* < 0.001.

## Glossary

LSCC: Laryngeal squamous cell carcinoma
qRT-PCR: Quantitative real-time reverse transcription polymerase chain reaction
FBS: Fetal Bovine serum
OD: Optical density
GO: Gene Ontology
KEGG: Kyoto Encyclopedia of Genes and Genomes
DEGs: Differentially expressed genes
GAPDH: Glyceraldehyde-3-phosphate dehydrogenase
PBS: Phosphate buffered saline
HNSC: Head and neck squamous cell carcinoma
EMT: Epithelial-mesenchymal transition
PPI: Protein-protein interaction
WHO: World Health Organization
SOX: SRY-related high-mobility group box
RBPs: RNA-binding proteins
DEPC: Diethyl pyrocarbonate
DMSO: Dimethyl sulfoxide
GEO: Gene Expression Omnibus
GSEA: Gene Set Enrichment Analysis
String: Search Tool for Rare or Non-redundant sequences
TCGA: The Cancer Genome Atlas Program
TIMER: Tumor Immune Estimation Resource
DepMap: Cancer Dependency Map
PDX: Patient-derived tumor xenograft
PTBP: polypyrimidine tract binding protein
PDXO: Patient-derived xenograft organoid
NCBI: National Center for Biotechnology Information.
