# Supplementary figures and images for: Transcription factor SOX4 promotes proliferation, invasion and lymphatic metastasis of laryngeal squamous cell carcinoma via PTBP2 activation
Source: Front Oncol. 2026 May 21;16:1829851. doi: 10.3389/fonc.2026.1829851 (PMC13233237; doi:10.3389/fonc.2026.1829851)

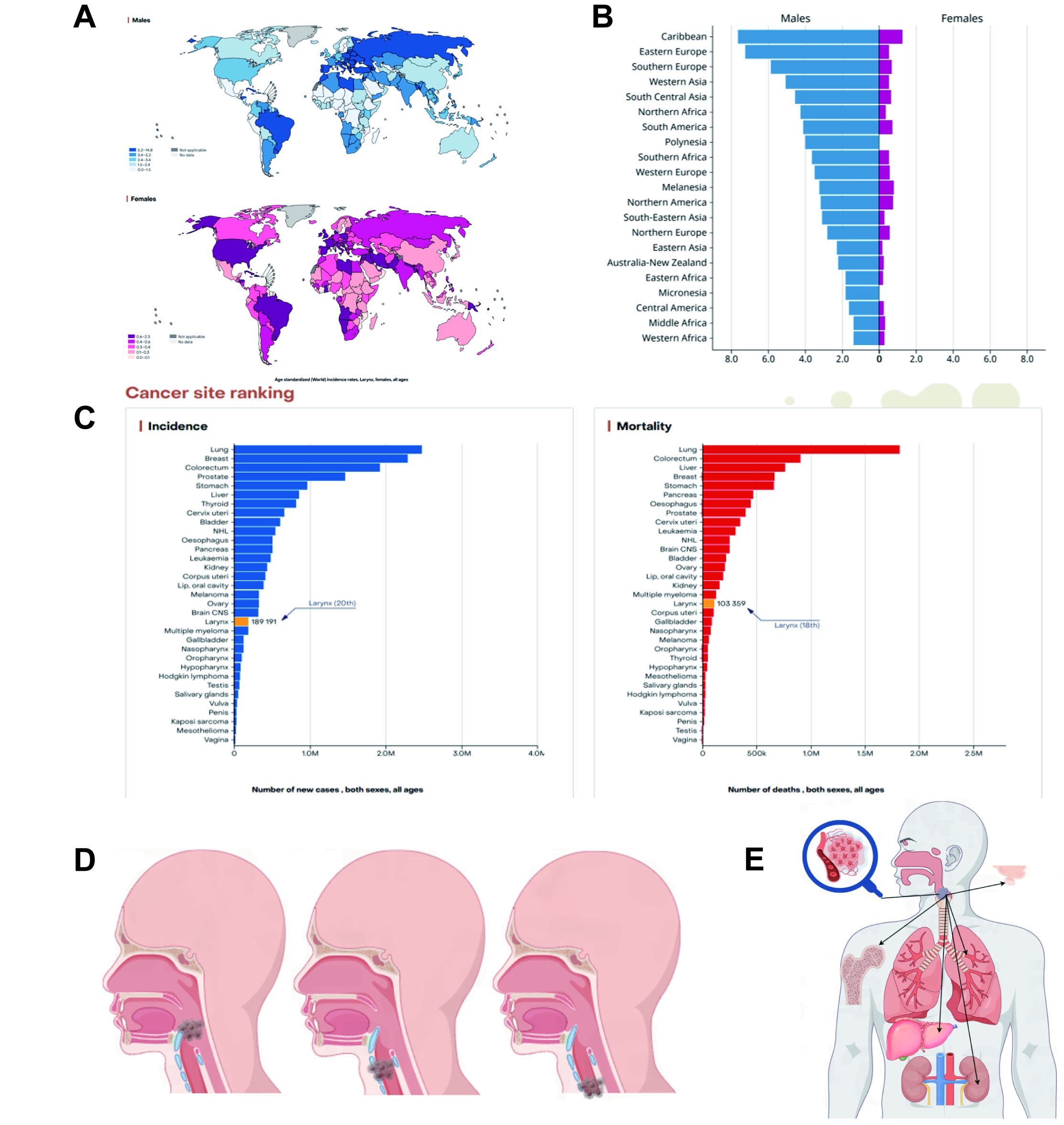

Supplement: Supplementary Figure 1 — Statistical data, clinical staging and metastatic characteristics of laryngeal cancer in 2026 by World Health Organization (WHO) global cancer (A) Global prevalence of laryngeal cancer in China; (B) Incidence rate and distribution of laryngeal cancer among men and women in various regions of the world; (C) Incidence rate and mortality rate of laryngeal cancer among tumors in the whole body; (D) Clinical common laryngeal cancer subtypes; and (E) Clinical common metastatic sites of laryngeal cancer. [file Image1.tif]

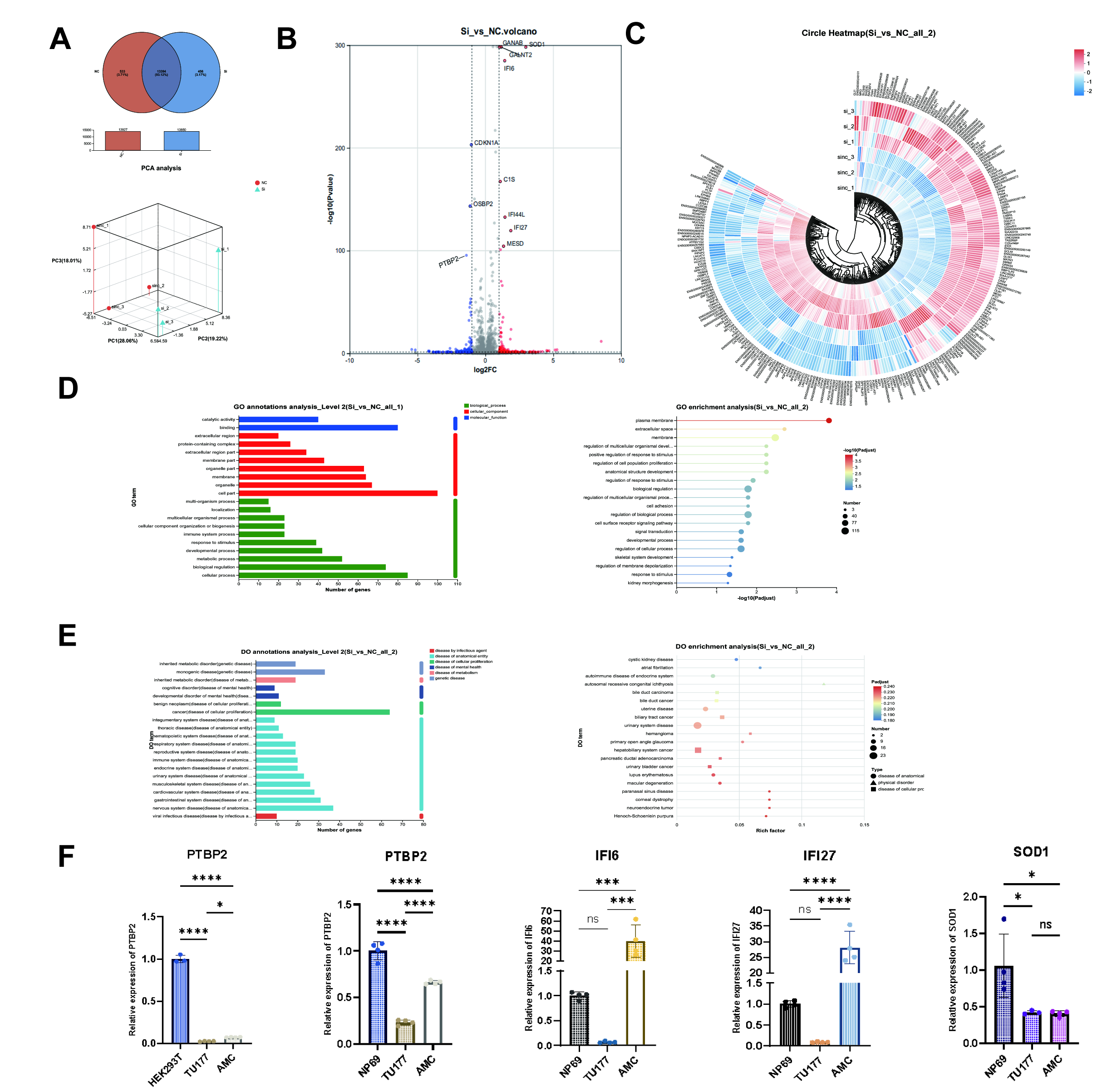

Supplement: Supplementary Figure 2 — SOX4 downstream target gene screening and validation (A) PCA plots of cellular transcriptome sequencing and differential gene Venn diagrams after knockdown of SOX4; (B, C) Volcano plots and heatmaps showing differential differentially differentiated genes with significantly up-regulated and down-regulated expression; (D) Bar graphs and bar graphs showing GO database annotation analyses and enrichment analyses of pathways, respectively; (E) Bar graphs and bar graphs showing DO database annotation analyses and enrichment analyses of pathways, respectively; (F) Bar graphs showing the cellular expression of PTBP2, IFI6, and IFI27 after knockdown of SOX4. *P < 0.05, ** P < 0.01, *** P < 0.001. [file Image2.tif]

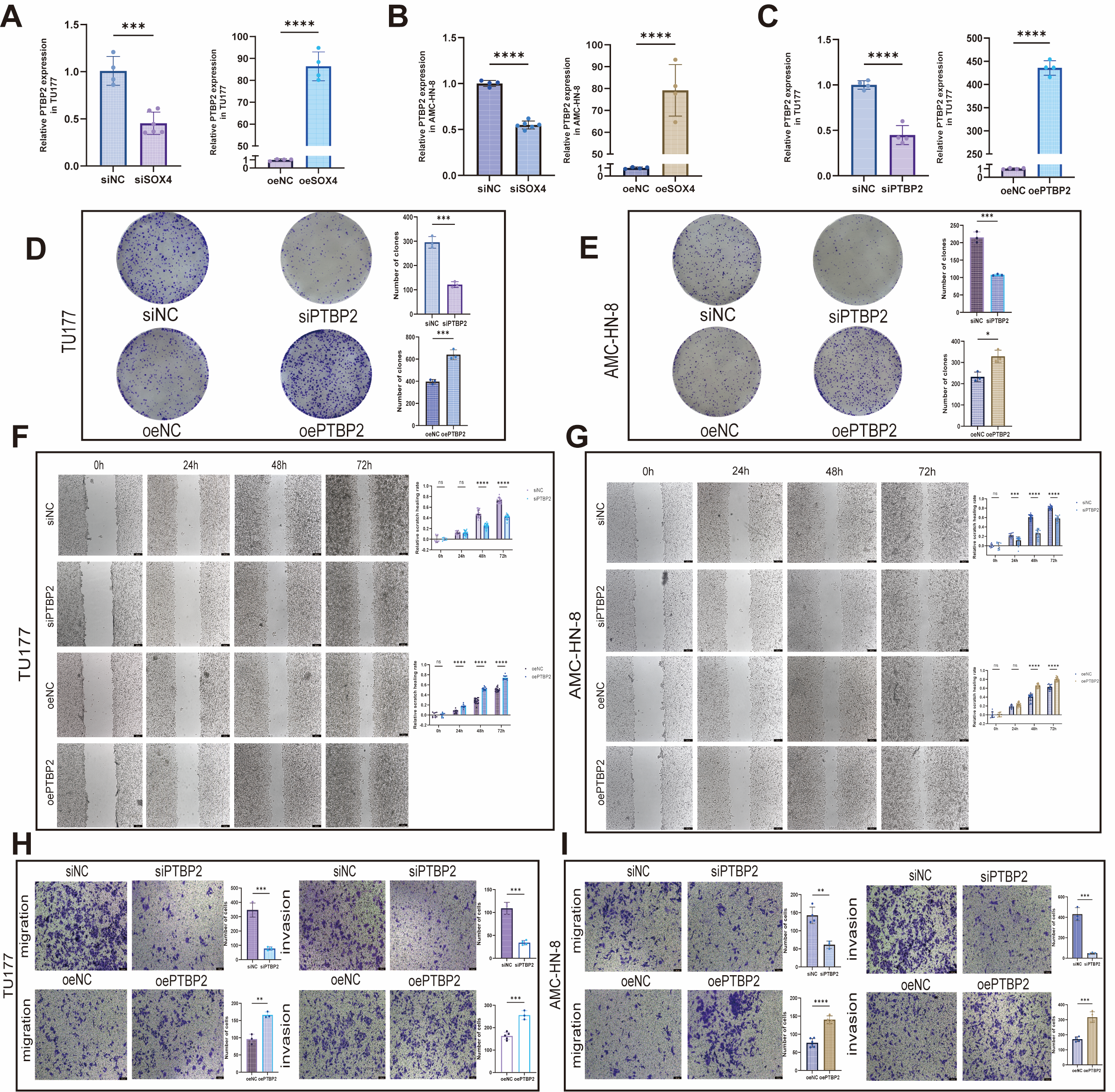

Supplement: Supplementary Figure 3 — PTBP2 expression in laryngeal squamous carcinoma cells and its effect on cell proliferation, migration and invasion ability (A) Bar graph showing the expression level of PTBP2 in TU177 after knockdown/overexpression of SOX4; (B) Bar graph showing the expression level of PTBP2 in AMC-HN-8 after knockdown/overexpression of SOX4; (C) Bar graph showing the expression efficiency of PTBP2 after knockdown/overexpression of PTBP2 in TU177; (D, E) Results of the clone formation assay showing the proliferation changes in laryngeal cancer cells after knockdown/overexpression of SOX4; (F, G) Bar graph showing the cell scratch healing rate after knockdown and overexpression of PTBP2; (H, I) Transwell assay showing that PTBP2 regulates the migration and invasion ability of laryngeal cancer cells. *P < 0.05, ** P < 0.01, *** P < 0.001. [file Image3.tif]
